# Preparedness of undergraduate health students to provide women-specific epilepsy care: knowledge gaps and stigmatizing attitudes in Saudi Arabia

**DOI:** 10.3389/fneur.2026.1983041

**Published:** 2026-10-30

**Authors:** Fahad Alzahrani, Yasir Alharbi, Yazeed Almuzaini, Abdulaziz Abdulhakeem, Roba Almughadhwi, Deema Aljohani, Abdulaziz Alhujayri, Abdulrahman Alsaadi, Alaa M. Alhammad, Bandar D. Alrehaili, Faris S. Alnezary

**Affiliations:** 1 Department of Pharmacy Practice, College of Pharmacy, Taibah University, Madinah, Saudi Arabia; 2 Scientific Research Unit, College of Pharmacy, Taibah University, Madinah, Saudi Arabia; 3 Pharmacology and Toxicology Department, College of Pharmacy, Taibah University, Madinah, Saudi Arabia

**Keywords:** epilepsy stigma, health professions education, health professions students, reproductive health, women with epilepsy

## Abstract

**Background:**

Women with epilepsy have distinct reproductive, clinical, and psychosocial healthcare needs that require women-specific care. This study assessed the preparedness of undergraduate health students by examining their knowledge, attitudes, and stigmatizing beliefs.

**Methods:**

A cross-sectional study was conducted among 339 undergraduate health students at Taibah University, Saudi Arabia, using a structured self-administered questionnaire. Multiple linear regression examined the factors associated with total knowledge score, while the ≥60% knowledge benchmark was retained for descriptive and exploratory sensitivity analyses.

**Results:**

The mean knowledge score was 7.37 ± 2.96 out of 16 (46.1%), and 23.6% met the ≥60% benchmark. Knowledge gaps were most evident in reproductive health, hormonal influences, fertility, antiseizure medication safety, and contraceptive counseling topics. Attitudes were generally supportive, although stigma persisted regarding marriage, parenting, and close relationships. Male gender and earlier academic year were associated with lower adjusted knowledge scores, whereas field of study, epilepsy history, formal education, and recent training were not significantly associated with knowledge.

**Conclusion:**

Undergraduate health students demonstrated substantial knowledge gaps in women-specific epilepsy care, despite generally supportive attitudes. Reviewing curricular coverage and addressing identified gaps in reproductive health, medication safety, contraception, pregnancy-related care, and stigma may enhance preparedness for evidence-based, patient-centered care. The threshold-based logistic sensitivity analysis should be considered exploratory because of the low events-per-parameter ratio.

## Introduction

Epilepsy is a common chronic neurological disorder that affects more than 50 million people worldwide and contributes substantially to disability and reduced quality of life. Women with epilepsy face additional clinical, reproductive, and psychosocial challenges across their lifespan, including contraception, fertility, pregnancy planning, antiseizure medication safety, hormonal influences, breastfeeding, and mental health concerns (1–3). They may also face stigma that affects their education, employment, relationships, family planning, and engagement with healthcare (4, 5). These issues distinguish women-specific epilepsy care from general epilepsy management and require healthcare professionals to provide appropriate counseling and coordinated support for women with epilepsy.

Undergraduate health professions education provides an important opportunity to develop the knowledge, communication skills, and professional attitudes required for such care in the future. This is particularly relevant because women with epilepsy may receive care from physicians, pharmacists, nurses, rehabilitation professionals, and other healthcare providers. The International League Against Epilepsy (ILAE) competency-based educational framework emphasizes competencies in counseling and clinical management, including patient and family education and the care of special populations, such as women of childbearing age (6). Inadequate preparation in these areas may contribute to misconceptions, missed counseling opportunities, and inconsistent care delivery.

Previous studies among healthcare and university students have identified variable epilepsy knowledge and persistent misconceptions and stigma, including concerns related to marriage, employment, parenting, and social participation (7–11). However, these studies have predominantly assessed general epilepsy knowledge and attitudes, with substantially less attention to women-specific issues such as reproductive health, hormonal influences, pregnancy, contraception, and antiseizure medication safety (3, 12–14). Consequently, it remains unclear whether undergraduate health students are adequately prepared to address the specific needs of women with epilepsy.

In Saudi Arabia, epilepsy remains associated with cultural misconceptions and social stigma (14, 15). However, evidence regarding undergraduate preparedness for women-specific epilepsy care is limited. Unlike previous studies focused mainly on general epilepsy knowledge, the present study evaluates reproductive, hormonal, medication safety, pregnancy-related, attitudinal, and stigma-related domains within a multidisciplinary undergraduate health student population. Therefore, this study assessed students’ knowledge, attitudes, and stigmatizing beliefs regarding women-specific epilepsy care and examined factors associated with total knowledge scores to identify areas that may inform future educational interventions.

## Materials and methods

### Study design and setting

We conducted a cross-sectional study from September 5 to November 16, 2025, to evaluate the preparedness of undergraduate health students to care for women with epilepsy. The study was conducted at Taibah University in Madinah, Saudi Arabia, a large public university offering undergraduate programs in medicine, pharmacy, dentistry, nursing, applied medical sciences, and medical rehabilitation. This study was designed and reported in accordance with the Strengthening the Reporting of Observational Studies in Epidemiology (STROBE) guidelines for cross-sectional studies (16).

### Participants

Eligible participants were undergraduate students enrolled in one of the health-related colleges at Taibah University, including the colleges of medicine, pharmacy, dentistry, nursing, applied medical sciences, and medical rehabilitation. Students were eligible if they were at least 18 years of age, currently enrolled in an eligible program, and able to provide electronic informed consent. Information regarding a personal diagnosis of epilepsy or epilepsy in an immediate family member was also collected because previous exposure to epilepsy may influence the knowledge and attitudes toward women with epilepsy.

### Recruitment and sample size

Participants were recruited using convenience and snowball sampling. An invitation containing a link to the online questionnaire was distributed through official university WhatsApp groups used for student communications. Participants were encouraged to share the survey with their eligible classmates to maximize participation across different health disciplines. We estimated the sample size using the Raosoft^®^ online calculator based on an estimated eligible student population of approximately 6,422, assuming a 95% confidence level, 5% margin of error, and a conservative response distribution of 50%. The minimum required sample size was 363. The final analytic sample comprised 339 participants, representing 93.4% of the target. We considered the implications of not reaching the target sample size, particularly for multivariable analyses, when interpreting the findings.

Approximately 820 students were invited to participate in the study, of whom 531 responded to the questionnaire. After excluding 192 responses for ineligibility or incomplete data, we included 339 eligible participants with complete questionnaire responses in the final analysis (Figure 1). To acknowledge the participants’ time, respondents could enter a prize draw. Entry into the draw was voluntary and not required for participation in this study. Participants were informed that they could decline or withdraw from the study without penalty. Participants who wished to enter the prize draw provided their university email address. Email addresses were removed from the dataset before analysis and were not used in the study analyses.

**Figure 1:**
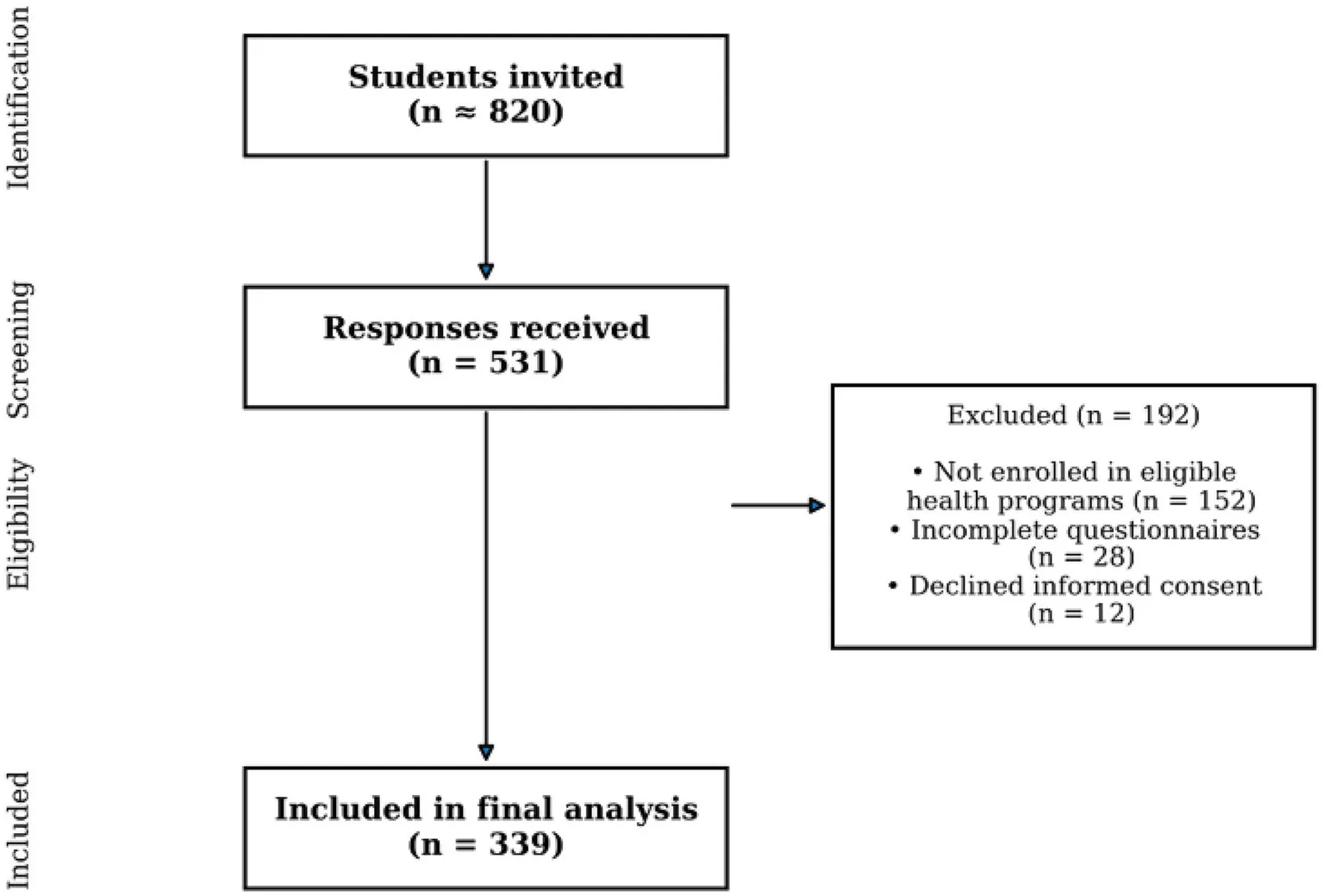
Flow diagram of student recruitment and selection.

### Questionnaire development

Data were collected using a structured, self-administered questionnaire developed through an iterative process informed by previously published instruments evaluating epilepsy-related knowledge, attitudes, and stigma among healthcare students and professionals (9, 17). Particular emphasis was placed on women-specific aspects of epilepsy care identified in recent international recommendations and the published literature, including reproductive health, pregnancy, antiseizure medication safety, contraception, hormonal influences on seizure control, and epilepsy-related stigma.

To establish content validity, three academic experts with experience in neurology, pharmacy, and clinical psychology independently reviewed the preliminary questionnaire. The experts evaluated each item for clinical relevance, clarity, cultural appropriateness, and content comprehensiveness. After consensus discussions, the authors made minor wording changes to improve readability while preserving each item’s original meaning. The final questionnaire was administered electronically in English using Google Forms.

### Study measures

The questionnaire consisted of four sections.

#### Demographic and educational characteristics

Participants reported their age, gender, academic discipline, academic year, prior exposure to epilepsy, prior education related to women with epilepsy, prior women-specific epilepsy training, and interest in furthering their education on the subject.

#### Knowledge of women-specific epilepsy care

Knowledge was assessed using 16 items with three response options (“Yes,” “No,” and “I do not know”) in the administered questionnaire. To identify clinically relevant educational gaps, the knowledge items were organized into three predefined content domains: general epilepsy knowledge (eight items), women-specific reproductive and hormonal health (six items), and medication safety and pregnancy-related issues (two items). Correct responses received 1 point, while incorrect and “I do not know” responses received 0 points. Total scores ranged from 0 to 16, with higher scores indicating greater knowledge. The total knowledge score was treated as a continuous measure in the primary analyses. For descriptive comparability with previous epilepsy education studies, the prespecified ≥60% threshold was retained as a secondary benchmark; however, this cutoff has not been validated as a measure of clinical competence. Because the score comprised 16 discrete items, the ≥60% criterion corresponded to a score of ≥10/16 (15, 17, 18). For publication, Supplementary File S1 presents the knowledge items as declarative statements with equivalent “True,” “False,” and “I do not know” labels to facilitate interpretation of the scoring key; the underlying item content and scoring were unchanged.

#### Attitudes toward women with epilepsy

Attitudes toward women with epilepsy were assessed using eight statements rated on a five-point Likert scale ranging from strongly disagree (1) to strongly agree (5). The items addressed perceived capabilities, social participation, women-specific challenges, and family-related issues. Item-level responses were descriptively summarized.

#### Perceived stigma

We assessed stigma-related perceptions using 10 statements rated on a five-point Likert scale. The items addressed both personal stigmatizing beliefs and perceptions of societal stigma. We summarized item-level responses descriptively and then examined the dimensional structure of the stigma items using exploratory factor analysis. Because the analysis identified distinct personal and perceived societal stigma dimensions, the 10 items were not interpreted as a single unidimensional stigma scale. A publication-formatted version of the questionnaire is provided in Supplementary File S1.

### Content validity and reliability

The questionnaire underwent content validation and reliability testing before implementation. Test–retest reliability was evaluated in a convenience sample of 27 graduate students who were not eligible to participate in the main study and completed the questionnaire on two occasions separated by 2 days (19). The test–retest correlation was *r* = 0.93 (95% CI 0.89–0.97; *p* < 0.001), indicating a strong short-term test–retest correlation. Internal consistency of the attitude and stigma item sets was assessed using Cronbach’s alpha (20). The eight attitude items demonstrated acceptable overall internal consistency (*α* = 0.776), and the 10 stigma items demonstrated good overall internal consistency (*α* = 0.823); however, subsequent exploratory factor analysis indicated a multidimensional structure. For the dichotomously scored knowledge items, the Kuder–Richardson 20 (KR-20) coefficient was 0.662 overall, 0.398 for the eight-item general epilepsy knowledge domain, and 0.560 for the six-item women-specific reproductive and hormonal domain. For the two-item medication-safety domain, the inter-item correlation was 0.329 and the Spearman–Brown coefficient was 0.496. Accordingly, the knowledge domains were treated as descriptive content categories rather than validated unidimensional subscales.

A *post hoc* exploratory factor analysis (EFA) of the 10 stigma items was conducted using principal axis factoring with Direct Oblimin rotation, as the underlying dimensions were expected to be correlated. Sampling adequacy was assessed using the Kaiser–Meyer–Olkin (KMO) measure and Bartlett’s test of sphericity. Factor retention was guided by eigenvalues >1 and the inspection of the scree plot. Pattern-matrix loadings ≥0.30 were considered for factor interpretation, and items with loadings ≥0.30 on more than one factor were considered to be cross-loading. Internal consistency was subsequently assessed separately for the items loading clearly on each factor using Cronbach’s alpha (α).

### Statistical analysis

All analyses were performed using IBM SPSS Statistics (version 27; IBM Corp., Armonk, NY, United States). Categorical variables were summarized using frequencies and percentages, while total knowledge scores were summarized using the mean, standard deviation (SD), median, range, and score distribution. Because the prespecified ≥60% threshold has not been validated as a measure of clinical competency, the total knowledge score (0–16) was considered the primary continuous outcome.

Multiple linear regression was used to examine factors associated with total knowledge score, adjusting for gender, academic year, field of study, personal or immediate family history of epilepsy, formal education on women-specific epilepsy, and recent women-specific epilepsy training. Covariates were selected *a priori* based on conceptual relevance. Age was not included alongside academic year because of their overlap, while interest in further training was not included because it may reflect perceived educational need rather than an antecedent characteristic. Unstandardized coefficients (B), 95% confidence intervals (CIs), and *p*-values were reported. Reference categories were female gender, fifth academic year or higher, applied medical sciences, “I’m not sure” for personal or immediate family history of epilepsy, “Yes, briefly” for formal education, and “Never attended” for recent training. Overall effects of categorical variables with more than two levels were evaluated using Type III tests.

Regression assumptions were assessed using the histogram and normal P–P plot of standardized residuals, the standardized residual-versus-predicted plot, the Durbin–Watson statistic, studentized deleted residuals, Cook’s distance, leverage values, tolerance, and variance inflation factor (VIF) statistics.

Bivariate associations with meeting the ≥60% benchmark were examined using chi-square or Fisher’s exact tests, as appropriate. *P*-values were adjusted using the Benjamini–Hochberg false discovery rate (FDR) procedure. The ≥60% benchmark was operationalized as ≥10/16 (62.5%), with additional sensitivity thresholds of ≥9/16 (56.3%) and ≥11/16 (68.8%), respectively.

Binary logistic regression using the ≥10/16 benchmark was conducted as an exploratory secondary sensitivity analysis. To limit model complexity, the model included gender, academic year, personal or immediate family history of epilepsy, formal education, and recent training; it did not include field of study. Adjusted odds ratios (aORs), 95% CIs, and *p*-values were reported. Model performance was assessed using the likelihood-ratio chi-square test, Cox and Snell *R*^2^, Nagelkerke *R*^2^, Hosmer–Lemeshow test, and area under the receiver operating characteristic curve (AUC). Because only 80 participants met the benchmark relative to 13 estimated parameters, the model was interpreted cautiously.

Potential non-response bias was explored by comparing the first quartile of eligible respondents (*n* = 84) with the final quartile (*n* = 85). Gender, academic year, and field of study were compared using chi-square tests, and total knowledge scores were compared using an independent-samples *t*-test. Except for FDR-adjusted bivariate analyses, all tests were two-sided with *p* < 0.05 considered statistically significant.

### Ethical approval

The College of Pharmacy, Taibah University Research Ethics Committee, Madinah, Saudi Arabia, approved this study (approval no. COPTU-REC-106-20240905). All participants provided electronic informed consent before completing the questionnaires. Participation was voluntary. University email addresses provided solely for the optional prize draw were removed before analysis and were not included in the analytic dataset, which contained no direct personal identifiers. All procedures complied with the ethical principles of the Declaration of Helsinki.

## Results

### Participant characteristics

Approximately 820 students were invited to participate in the study, and 531 responses were received. After excluding ineligible and incomplete responses, 339 eligible undergraduate health students were included in the final analysis (Figure 1). Most participants were aged 18–21 years (62.2%), female (70.8%), and enrolled in the fifth academic year or higher (26.8%). Medical Rehabilitation Sciences represented the largest academic discipline (26.5%), followed by pharmacy (22.4%). Most participants reported no personal or immediate family history of epilepsy (79.6%). Regarding formal education on women-specific epilepsy during their studies, 32.7% reported brief exposure, 11.8% reported extensive exposure, and 55.5% selected “I’m not sure” regarding whether such content had been covered. More than half (52.5%) had never attended dedicated training on women-specific epilepsy issues, while 83.5% expressed interest in receiving further education on epilepsy and women’s health (Table 1).

**Table 1:** Demographic and educational characteristics of participants (*N* = 339).

Characteristic	Category	*n*	%
Age	18–21	211	62.2
	22–25	115	33.9
	26–29	13	3.8
Gender	Male	99	29.2
	Female	240	70.8
Academic year	First year	47	13.9
	Second year	69	20.4
	Third year	68	20.1
	Fourth year	64	18.9
	Fifth year or higher	91	26.8
Field of study	Pharmacy	76	22.4
	Medicine	25	7.4
	Dentistry	54	15.9
	Nursing	52	15.3
	Applied medical sciences	42	12.4
	Medical Rehabilitation Sciences	90	26.5
Personal or immediate family history of epilepsy	No	270	79.6
	Yes	48	14.2
	I’m not sure	21	6.2
Formal education on women-specific epilepsy during studies	I’m not sure	188	55.5
	Yes, extensively	40	11.8
	Yes, briefly	111	32.7
Recent training on women-specific epilepsy issues	Never attended	178	52.5
	Within the past year	49	14.5
	1–3 years ago	35	10.3
	More than 3 years ago	14	4.1
	I’m not sure	63	18.6
Interest in epilepsy and women’s health training	No	56	16.5
	Yes	283	83.5

As a proxy for potential non-response bias, we compared the first quartile of eligible respondents (*n* = 84) with the final quartile (*n* = 85). No significant differences were observed in gender distribution [*χ*^2^(1) = 2.39, *p* = 0.123], field of study [*χ*^2^(5) = 9.72, *p* = 0.083], or total knowledge scores [7.62 ± 2.75 vs. 7.22 ± 3.42; *t*(167) = 0.83, *p* = 0.409]. However, academic-year distribution differed significantly between early and late responders [*χ*^2^(4) = 23.65, *p* < 0.001], with early responders more frequently enrolled in the fifth year or higher.

### Educational preparedness for women-specific epilepsy care

Overall knowledge was limited, with a mean score of 7.37 ± 2.96 out of 16 (46.1%), a median of 7.0, and an observed range of 0–15. Using the predefined descriptive benchmark of ≥10/16, 80 participants (23.6%) met the ≥60% criterion. Sensitivity analyses using alternative thresholds showed that 120 participants (35.4%) scored ≥9/16 (56.3%), whereas 56 participants (16.5%) scored ≥11/16 (68.8%). Overall, 219 participants (64.6%) scored at or below 8/16 (50%). Supplementary Figure S1 shows the overall distribution of knowledge scores. For general knowledge of epilepsy, participants had the highest correct response rates for recognizing convulsions (76.1%) and loss of consciousness (74.0%) as symptoms. In addition, 66.7% recognized that epilepsy could be controlled, and 61.7% knew that epilepsy could be hereditary. Lower correct response rates were observed for identifying epilepsy as not being a mental illness (41.0%), recognizing that epilepsy is not necessarily curable with modern medications (25.4%), identifying epilepsy surgery as a treatment option (24.5%), and correctly rejecting uncontrolled shouting as a typical symptom (22.7%).

Knowledge of women-specific reproductive health was generally low. Although 75.2% of respondents recognized that women with epilepsy require specialized care during pregnancy, fewer than half correctly answered questions concerning postpartum depression (47.5%), hormonal and reproductive influences (43.7%), reproductive health (39.2%), and fertility issues (29.8%). Only 10.3% correctly identified that epilepsy was not more prevalent in women. Regarding medication safety, 59.9% of respondents correctly recognized that some antiseizure medications might increase the risk of congenital malformations, whereas only 39.5% correctly identified the potential interactions between antiseizure medications and hormonal contraceptives (Table 2).

**Table 2:** Knowledge of women-specific epilepsy care among undergraduate health students (*N* = 339).

Domain/knowledge item	Correct response	Correct response, *n* (%)
General knowledge about epilepsy		
Epilepsy can be hereditary	True	209 (61.7)
Epilepsy is a form of mental illness	False	139 (41.0)
Convulsions are a symptom of epilepsy	True	258 (76.1)
Loss of consciousness is a symptom of epilepsy	True	251 (74.0)
Uncontrolled shouting is a symptom of epilepsy	False	77 (22.7)
Epilepsy can be controlled	True	226 (66.7)
Epilepsy is curable with modern medications	False	86 (25.4)
Epilepsy surgery is a treatment option	True	83 (24.5)
Women-specific reproductive health		
Epilepsy is more common in women	False	35 (10.3)
Epilepsy can affect reproductive health	True	133 (39.2)
Epilepsy increases infertility risk	True	101 (29.8)
Women with epilepsy require specialized care during pregnancy	True	255 (75.2)
Women with epilepsy have an increased risk of postpartum depression	True	161 (47.5)
Hormonal and reproductive factors influence epilepsy	True	148 (43.7)
Medication safety		
Some antiseizure medications may increase the risk of congenital malformations	True	203 (59.9)
Antiseizure medications may reduce the effectiveness of hormonal contraceptives	True	134 (39.5)
Overall knowledge score		7.37 ± 2.96 out of 16 (46.1%)

Data are presented as *n* (%) unless otherwise specified. Overall knowledge is presented as the mean ± standard deviation (SD).

### Attitudes toward women with epilepsy

Participants generally expressed supportive attitudes toward the capabilities and social roles of women with epilepsy. Most participants agreed that women with epilepsy should be able to receive education (82.9%), perform daily activities (80.2%), be employed (80.8%), marry (83.5%), and have children (80.8%). In addition, 65.8% of the respondents agreed that women with epilepsy should participate in sports, and 65.8% recognized gender-specific challenges in epilepsy management. However, 49.6% of the respondents believed that families experience considerable distress when the mother has epilepsy, indicating that concerns about the broader psychosocial impact of epilepsy persisted despite generally supportive attitudes (Table 3).

**Table 3:** Students’ attitudes toward women with epilepsy (*N* = 339).

Statement	Disagree *n* (%)	Neutral *n* (%)	Agree *n* (%)	Mean
There are gender-specific challenges in managing epilepsy in women	17 (5.0)	99 (29.2)	223 (65.8)	3.95
Women with epilepsy can receive education	10 (2.9)	48 (14.2)	281 (82.9)	4.43
Women with epilepsy can perform daily activities	11 (3.2)	56 (16.5)	272 (80.2)	4.29
Women with epilepsy should participate in sports	21 (6.2)	95 (28.0)	223 (65.8)	3.94
A woman with epilepsy can have a job	11 (3.2)	54 (15.9)	274 (80.8)	4.32
A woman with epilepsy can marry	12 (3.5)	44 (13.0)	283 (83.5)	4.42
Women with epilepsy can have children	11 (3.2)	54 (15.9)	274 (80.8)	4.34
There is distress in the family when the mother has epilepsy	51 (15.0)	120 (35.4)	168 (49.6)	3.57

### Perceived stigma

Participants recognized the substantial societal stigma surrounding epilepsy, and personal stigmatizing beliefs were evident in some domains. Most participants perceived epilepsy as being misunderstood by the general public (71.1%), associated with educational or employment barriers (62.2%), and linked to stereotypes and misconceptions (57.2%). Personal concerns were particularly evident in close relationships: 44.0% reported hesitancy about marrying someone with epilepsy, and 35.7% expressed concerns about the safety of children around someone with epilepsy. These findings suggest that supportive attitudes toward social participation may coexist with persistent personal and perceived societal stigma (Table 4).

**Table 4:** Students’ perceptions of epilepsy-related stigma (*N* = 339).

Statement	Disagree *n* (%)	Neutral *n* (%)	Agree *n* (%)	Mean
People with epilepsy are often viewed as less capable	71 (20.9)	65 (19.2)	203 (59.9)	3.55
I would feel uncomfortable socializing with someone who has epilepsy	182 (53.7)	69 (20.4)	88 (26.0)	3.57
Epilepsy is seen as a sign of weakness or lack of willpower	150 (44.2)	74 (21.8)	115 (33.9)	3.22
People with epilepsy are often discriminated against in the workplace	81 (23.9)	115 (33.9)	143 (42.2)	3.22
I would be hesitant to marry someone with epilepsy	82 (24.2)	108 (31.9)	149 (44.0)	2.76
Epilepsy is misunderstood by the general public	25 (7.4)	73 (21.5)	241 (71.1)	3.99
People with epilepsy are excluded from social activities	70 (20.6)	87 (25.7)	182 (53.7)	3.42
Concerned about the safety of children around someone with epilepsy	123 (36.3)	95 (28.0)	121 (35.7)	3.05
Epilepsy is associated with stereotypes and misconceptions	36 (10.6)	109 (32.2)	194 (57.2)	3.69
People with epilepsy face education/employment barriers	39 (11.5)	89 (26.3)	211 (62.2)	3.71

Exploratory factor analysis supported a two-factor structure for the stigma items [KMO = 0.854; Bartlett’s *χ*^2^(45) = 1056.29, *p* < 0.001]. The two factors accounted for 43.9% of the variance and were moderately correlated (*r* = 0.346). The first factor comprised items reflecting personal stigma, including discomfort with socializing, hesitancy to marry, concerns about children’s safety, and perceptions of epilepsy as weakness, and demonstrated acceptable internal consistency (Cronbach’s *α* = 0.794). The second factor comprised items reflecting perceived societal stigma, including public misunderstanding, social exclusion, stereotypes, and education or employment barriers, and demonstrated lower internal consistency (Cronbach’s *α* = 0.649). Two additional items concerning perceived capability and workplace discrimination showed cross-loadings and were interpreted cautiously (Supplementary Table S2).

Responses were originally recorded on a five-point Likert scale and are presented in three categories for descriptive reporting. The exploratory factor analysis of these items is presented in Supplementary Table S2.

### Bivariate associations with meeting the ≥60% knowledge benchmark

In bivariate analyses, meeting the ≥60% knowledge benchmark was associated with gender, academic year, personal or immediate family history of epilepsy, and formal education on women-specific epilepsy. After Benjamini–Hochberg FDR correction for multiple comparisons, all four associations remained statistically significant: gender (adjusted *p* = 0.048), academic year (adjusted *p* = 0.048), personal or immediate family history of epilepsy (adjusted *p* = 0.016), and formal education (adjusted *p* = 0.048). Age (adjusted *p* = 0.185), field of study (adjusted *p* = 0.498), recent women-specific epilepsy training (adjusted *p* = 0.123), and interest in further training (adjusted *p* = 0.787) were not significantly associated with meeting the ≥60% knowledge benchmark (Table 5).

**Table 5:** Bivariate associations between participant characteristics and meeting the ≥60% knowledge benchmark (*N* = 339).

Characteristic	Category	<60% *n* (%)	≥60% *n* (%)	*χ* ^2^	Raw *p*	FDR-adjusted *p*
Age	18–21	168 (79.6)	43 (20.4)	3.95	0.139	0.185
	22–25	83 (72.2)	32 (27.8)			
	26–29	8 (61.5)	5 (38.5)			
Gender	Male	84 (84.8)	15 (15.2)	5.53	0.019	0.048
	Female	175 (72.9)	65 (27.1)			
Academic year	First year	38 (80.9)	9 (19.1)	11.23	0.024	0.048
	Second year	57 (82.6)	12 (17.4)			
	Third year	55 (80.9)	13 (19.1)			
	Fourth year	51 (79.7)	13 (20.3)			
	Fifth year or higher	58 (63.7)	33 (36.3)			
Field of study	Medical Rehabilitation Sciences	68 (75.6)	22 (24.4)	4.83	0.436	0.498
	Pharmacy	57 (75.0)	19 (25.0)			
	Medicine	18 (72.0)	7 (28.0)			
	Dentistry	38 (70.4)	16 (29.6)			
	Nursing	41 (78.8)	11 (21.2)			
	Applied medical sciences	37 (88.1)	5 (11.9)			
Personal or immediate family history of epilepsy	No	217 (80.4)	53 (19.6)	12.15	0.002	0.016
	Yes	28 (58.3)	20 (41.7)			
	I’m not sure	14 (66.7)	7 (33.3)			
Formal education on women-specific epilepsy during studies	I’m not sure	155 (82.4)	33 (17.6)	8.61	0.013	0.048
	Yes, extensively	27 (67.5)	13 (32.5)			
	Yes, briefly	77 (69.4)	34 (30.6)			
Recent training on women-specific epilepsy issues	Never attended	145 (81.5)	33 (18.5)	8.45	0.077	0.123
	Within the past year	31 (63.3)	18 (36.7)			
	1–3 years ago	24 (68.6)	11 (31.4)			
	More than 3 years ago	11 (78.6)	3 (21.4)			
	I’m not sure	48 (76.2)	15 (23.8)			
Interest in epilepsy and women’s health training	No	42 (75.0)	14 (25.0)	0.07	0.787	0.787
	Yes	217 (76.7)	66 (23.3)			

FDR = Benjamini–Hochberg false discovery rate adjustment applied to the eight bivariate comparisons.

### Multivariable associations with total knowledge score

The multivariable linear regression model examining factors associated with the total knowledge score was statistically significant [*F*(18, 320) = 3.18, *p* < 0.001] and explained 15.2% of the variance in knowledge scores (*R*^2^ = 0.152; adjusted *R*^2^ = 0.104). The regression diagnostics did not indicate any major violations of the model assumptions. The standardized residual histogram and normal P–P plot were approximately consistent with normality, and the residual-versus-predicted plot showed no marked evidence of heteroscedasticity or systematic model misspecification. The Durbin–Watson statistic was 1.988. The studentized deleted residuals ranged from −2.764 to 2.609, Cook’s distances ranged from 0.000 to 0.048, and the maximum centered leverage value was 0.151, with no observations showing evidence of undue influence. No concerning multicollinearity was identified, with tolerance values ranging from 0.336–0.916 and VIF values ranging from 1.092–2.976. After adjusting for academic year, field of study, personal or immediate family history of epilepsy, formal education, and recent women-specific epilepsy training, male students had significantly lower knowledge scores than female students (*B* = −1.44, 95% CI: −2.13 to −0.75; *p* < 0.001). The academic year was also significantly associated with the total knowledge score (*p* = 0.009). Compared with students in the fifth year or higher, those in the first (*B* = −1.35, 95% CI: −2.40 to −0.30; *p* = 0.012), second (*B* = −1.49, 95% CI: −2.45 to −0.53; *p* = 0.002), third (*B* = −1.52, 95% CI: −2.47 to −0.58; *p* = 0.002), and fourth years (*B* = −1.03, 95% CI: −1.96 to −0.09; *p* = 0.032) had significantly lower mean knowledge scores. Field of study (*p* = 0.332), personal or immediate family history of epilepsy (*p* = 0.097), formal education on women-specific epilepsy (*p* = 0.099), and recent women-specific epilepsy training (*p* = 0.976) were not significantly associated with the total knowledge score after adjustment (Table 6). In the exploratory sensitivity analysis, meeting the ≥60% knowledge benchmark was examined as a binary outcome using the reduced adjustment set described above. The logistic regression model was statistically significant [*χ*^2^(13) = 37.92, *p* < 0.001; Nagelkerke *R*^2^ = 0.159], with an AUC of 0.720. The Hosmer–Lemeshow goodness-of-fit test was not significant [*χ*^2^(8) = 8.02, *p* = 0.431], indicating no evidence of poor model fit. Because only 80 participants met the ≥60% benchmark relative to 13 estimated parameters (approximately 6.2 events per parameter), threshold-based logistic regression was considered exploratory, and its estimates were interpreted cautiously (Supplementary Table S1).

**Table 6:** Multivariable linear regression analysis of factors associated with total knowledge score (*N* = 339).

Variable	Category	*B*	SE	95% CI	*p*-value	Overall *p*-value
Gender	Male	−1.436	0.350	−2.125 to −0.747	<0.001	<0.001*
	Female	reference				
Academic year	First year	−1.352	0.534	−2.402 to −0.303	0.012	0.009*
	Second year	−1.486	0.487	−2.445 to −0.527	0.002	
	Third year	−1.524	0.482	−2.473 to −0.576	0.002	
	Fourth year	−1.027	0.477	−1.964 to −0.089	0.032	
	Fifth year or higher	reference				
Field of study	Medical Rehabilitation Sciences	0.845	0.540	−0.218 to 1.908	0.119	0.332
	Pharmacy	1.038	0.567	−0.077 to 2.154	0.068	
	Medicine	0.483	0.729	−0.951 to 1.917	0.508	
	Dentistry	1.224	0.601	0.041 to 2.407	0.043	
	Nursing	0.439	0.599	−0.739 to 1.618	0.464	
	Applied medical sciences	reference				
Personal or immediate family history of epilepsy	No	−0.016	0.653	−1.301 to 1.268	0.980	0.097
	Yes	0.955	0.751	−0.523 to 2.432	0.205	
	I’m not sure	reference				
Formal education on women-specific epilepsy	I’m not sure	−0.831	0.400	−1.617 to −0.044	0.039	0.099
	Yes, extensively	0.077	0.543	−0.991 to 1.145	0.887	
	Yes, briefly	reference				
Recent women-specific epilepsy training	Within the past year	−0.272	0.528	−1.310 to 0.766	0.607	0.976
	I’m not sure	−0.130	0.424	−0.963 to 0.703	0.759	
	1–3 years ago	0.096	0.588	−1.060 to 1.252	0.870	
	More than 3 years ago	0.029	0.844	−1.633 to 1.690	0.973	
	Never attended	reference				

B = unstandardized regression coefficient; SE = standard error; CI = confidence interval. Overall *p*-values are from Type III tests of between-subjects effects; category-specific *p*-values are from parameter estimates. Model: *F*(18, 320) = 3.184, *p* < 0.001, *R*^2^ = 0.152, adjusted *R*^2^ = 0.104. Reference categories are indicated in the table. *Statistically significant overall Type III effect at *p* < 0.05.

## Discussion

This study identified substantial gaps in undergraduate health students’ preparedness for women-specific epilepsy care. The mean knowledge score was 7.37 ± 2.96 out of 16 (46.1%), and only 23.6% of the participants met the prespecified ≥60% knowledge benchmark. Knowledge gaps were most evident in reproductive health, hormonal influences, antiseizure medication safety, fertility-related issues, and contraceptive counseling. In contrast, students generally expressed supportive attitudes toward the educational, occupational, marital, and parental roles of women with epilepsy, although stigmatizing beliefs persisted in close personal and family relationships. In the primary adjusted analysis, male gender and earlier academic year were associated with lower total knowledge scores, whereas the field of study, personal or immediate family history of epilepsy, formal education, and recent women-specific epilepsy training were not significantly associated with knowledge.

The limited knowledge observed is broadly consistent with previous research, although direct numerical comparisons should be made cautiously because the populations, instruments, scoring systems, and knowledge domains differ. In Palestine, Shawahna et al. (17) assessed practicing pharmacists using a 12-item women-specific epilepsy knowledge test and reported a median correct score of 53.8%, indicating that knowledge gaps may persist even after professional qualification. In Saudi Arabia, Alshahrani et al. (9) identified incomplete epilepsy knowledge among undergraduate health students, while Hakami et al. (15) reported persistent misconceptions among university students from health and non-health disciplines. These findings suggest that the gaps identified in the present study are not confined to undergraduate education, although our instrument’s women-specific focus limits direct comparison. This study extends the literature by examining reproductive health, hormonal influences, fertility, pregnancy, antiseizure medication safety, and contraceptive interactions within a multidisciplinary undergraduate population.

The largest deficiencies were in reproductive health and medication safety. Fewer than half of the participants correctly answered questions concerning fertility, hormonal influences, postpartum depression, and interactions between antiseizure medications and hormonal contraceptives. These areas are clinically important because healthcare professionals across disciplines may counsel women before conception, during pregnancy, and throughout their reproductive years (2, 3, 21). Current clinical guidance also emphasizes careful selection and monitoring of antiseizure medications during pregnancy to reduce maternal and fetal risks (22). Insufficient knowledge may contribute to missed counseling opportunities, inappropriate medication advice, and delayed referrals (3, 23). Knowledge gaps have also been reported among practicing healthcare professionals involved in perinatal epilepsy care, suggesting that these educational challenges may extend beyond undergraduate training (23). Current recommendations further emphasize that reproductive counseling should occur across the lifespan rather than being limited to pregnancy (3, 21).

A high proportion of “I don’t know” responses was also observed for several women-specific items, particularly those concerning reproductive health and contraceptive interactions. These responses may indicate uncertainty rather than firmly established misconceptions and, therefore, represent potentially modifiable educational gaps in the curriculum. Case-based learning, simulation, and repeated clinical exposure may help strengthen knowledge and communication skills (24, 25).

Attitudes were generally favorable; however, stigma persisted in close relationships. In the present study, 44.0% of participants reported hesitancy about marrying someone with epilepsy, and 35.7% expressed concern about the safety of children around someone with epilepsy. Marriage-related concerns have also been reported in a large Saudi survey, in which 44.8% of respondents objected to a close relative marrying a person with epilepsy (26). Similar patterns have been reported in Saudi Arabia and Turkey (15, 27, 28). The international literature summarized by the ILAE Task Force on Stigma in Epilepsy also demonstrates that epilepsy-related stigma remains evident across diverse sociocultural settings (10). The coexistence of generally supportive attitudes with stigma in marriage, parenting, and close relationships may reflect context-dependent or multidimensional stigma; however, it may also partly reflect differences in the constructs assessed by the attitude and stigma items. Sociocultural expectations surrounding marriage and family roles may shape responses in the Saudi context (14, 26); however, cultural and religious beliefs were not directly measured in this study, and their specific contribution should not be inferred from these data.

Male gender remained associated with lower knowledge scores after adjustment, with an estimated difference of 1.44 points on the 16-point knowledge scale, equivalent to approximately 9% of the total scale range. Although statistically significant, the educational significance of this difference is uncertain because no validated minimally important difference exists for this knowledge measure. The association may reflect differences in exposure to women’s health content, interest in the topic, or differential self-selection into the study rather than gender itself. Female students comprised 70.8% of the sample, and participants with greater interest in women’s health may have been more likely to participate. Effect modification by field of study or academic year was not examined because the resulting subgroup analyses would have increased model complexity and produced relatively small strata. In addition, prior coursework in women’s health was not measured directly; therefore, residual confounding by differential educational exposure cannot be excluded. The gender association should therefore be interpreted cautiously and confirmed in larger, multi-institutional studies.

Academic year was also significantly associated with knowledge, with students in the first through fourth years having lower adjusted scores than those in the fifth year or higher. This pattern is broadly consistent with studies reporting greater epilepsy knowledge among students with more advanced clinical training (9, 27) and may reflect cumulative exposure to clinical and epilepsy-related content. However, curriculum content and individual educational exposure were not directly measured, and the cross-sectional design does not establish that knowledge increases as students progress through their programs.

A personal or immediate family history of epilepsy was not significantly associated with the total knowledge score after adjustment, although an association was observed in the threshold-based sensitivity analysis. Because the questionnaire combined personal and immediate family history into a single item, their separate associations with knowledge could not be examined. This discrepancy also highlights the sensitivity of the findings to outcome categorization and supports the cautious interpretation of the ≥60% benchmark.

Formal education on women-specific epilepsy and recent training were not significantly associated with total knowledge score after adjustment. These findings should not be interpreted as evidence that education or training is ineffective, because the study did not assess the content, quality, duration, intensity, or timing of these exposures. Reverse causality also cannot be excluded; students who perceived knowledge gaps may have been more likely to seek additional training. Future studies should characterize educational exposure in greater detail and prospectively evaluate whether structured interventions improve knowledge and clinically relevant competencies.

Collectively, these findings support the need to strengthen women-specific epilepsy content in undergraduate health professions curricula. Reproductive health, antiseizure medication safety, contraception, pregnancy, and stigma could be addressed through interdisciplinary teaching, case-based learning, simulations, and patient-centered communication (3, 21, 25). Future educational interventions should assess not only exposure but also educational quality, knowledge retention, communication skills, and clinically relevant competencies.

### Educational implications

The present study did not include formal curriculum mapping; therefore, the extent, timing, and placement of epilepsy and reproductive health content within individual health professions programs could not be determined. Participant reports nevertheless suggest variable educational exposure: 11.8% reported extensive formal education on women-specific epilepsy, 32.7% reported brief exposure, and 55.5% selected “I’m not sure” regarding whether such content had been covered. These findings support reviewing existing curricular coverage and strengthening women-specific epilepsy education where gaps are identified.

Relevant learning outcomes could be aligned with the International League Against Epilepsy (ILAE) competency-based framework, particularly its counseling domain and emphasis on patient and family counseling, family life, and the management of special populations, including women of childbearing age (6). Rather than adding stand-alone modules, educators could integrate concise content into existing pharmacology, neurology, reproductive health, and clinical skills courses through case-based learning, simulation, and periodic reinforcement. Interprofessional education involving students from medicine, pharmacy, nursing, rehabilitation, and other health disciplines may also strengthen collaborative counseling, referral, medication-safety decisions, and patient-centered communication.

Competency development could be assessed using case-based examinations, objective structured clinical examinations (OSCEs), simulation-based assessments, and workplace-based assessments during clinical training. Sustainability may be supported through shared interdisciplinary resources, faculty development, integration into existing assessments, and periodic curriculum reviews. These approaches are consistent with the World Health Organization (WHO) Intersectoral Global Action Plan on Epilepsy and Other Neurological Disorders 2022–2031, which emphasizes strengthening health-workforce capacity, improving person-centered neurological care, and reducing stigma and discrimination (29). Future educational studies should include formal curriculum mapping and evaluate feasibility, knowledge retention, clinical application, and sustainability.

### Strengths and limitations

This study examined preparedness for women-specific epilepsy care across multiple health disciplines. However, the cross-sectional, single-university design and convenience and snowball sampling may have introduced selection and non-response bias and limited generalizability. Female participants comprised 70.8% of the sample, and differential self-selection related to interest in women’s health may have influenced the observed gender association in either direction. Early- and late-responders also differed by academic year, and because academic year was independently associated with knowledge, differential participation by academic seniority may have influenced the observed findings. The results should therefore not be assumed to represent students at other Saudi universities, and multi-institutional replication is warranted.

The final sample was below the prespecified target. Although regression diagnostics did not identify major assumption violations or influential observations, the primary model explained a modest proportion of variation in knowledge scores (*R*^2^ = 0.152; adjusted *R*^2^ = 0.104), and internal validation was not performed. Unmeasured factors, including prior coursework, motivation, language proficiency, socioeconomic circumstances, and other educational or cultural exposures, may therefore have influenced knowledge. Effect modification of the gender association by field of study or academic year was not examined, and prior coursework in women’s health was not directly measured. The secondary logistic analysis was exploratory because only 80 participants met the ≥60% benchmark relative to 13 estimated parameters, increasing the potential for overfitting and unstable estimates.

The knowledge items demonstrated modest overall internal consistency, with lower reliability within the predefined content domains; therefore, these domains should be interpreted as descriptive categories rather than as validated unidimensional subscales. Responses were self-reported and may have been affected by recall, social desirability, or acquiescence bias. No separate social desirability scale was administered. Although exploratory factor analysis suggested distinct personal and perceived societal stigma dimensions, the perceived societal stigma factor showed modest internal consistency (*α* = 0.649), and two items demonstrated cross-loadings. Therefore, this factor structure should be considered preliminary and requires confirmation in independent samples. Test–retest reliability was assessed in 27 graduate students over a two-day interval, which may have inflated the estimated test–retest correlation.

The questionnaire was administered in English only, so variation in language proficiency may have affected comprehension. Formal curriculum mapping was not conducted. In addition, the formal-education item did not include an explicit “No” response option; consequently, the “I’m not sure” category may have included both students who were uncertain about prior exposure and those who had not received such education. Personal and immediate family history of epilepsy was assessed as a combined item, preventing their separate evaluation. Finally, the cross-sectional design precludes causal inference, and the study assessed knowledge, attitudes, and stigma rather than observed clinical competence.

## Conclusion

Undergraduate health students exhibited substantial knowledge gaps in women-specific epilepsy care, particularly in reproductive health, medication safety, hormonal influences, and contraceptive counseling. Although attitudes were generally supportive, stigma persisted regarding marriage and parenting. Male gender and earlier academic years were associated with lower total knowledge scores, whereas the field of study, personal or immediate family history of epilepsy, formal education on women-specific epilepsy, and recent women-specific epilepsy training were not significantly associated after adjustment. Greater integration of clinically relevant education on reproductive care, antiseizure medication safety, contraceptive counseling, and stigma reduction into undergraduate curricula may help improve preparedness for evidence-based, patient-centered care; however, prospective educational studies are needed to evaluate the effectiveness of such approaches.

## Data Availability

The original contributions presented in the study are included in the article/Supplementary material, further inquiries can be directed to the corresponding author.
