# Supplementary material for: Preparedness of undergraduate health students to provide women-specific epilepsy care: knowledge gaps and stigmatizing attitudes in Saudi Arabia
Source: Front Neurol. 2026 Oct 30;17:1983041. doi: 10.3389/fneur.2026.1983041 (PMC13759284; doi:10.3389/fneur.2026.1983041)
Supplement: Supplementary file 1 [file Data_Sheet_1.pdf]

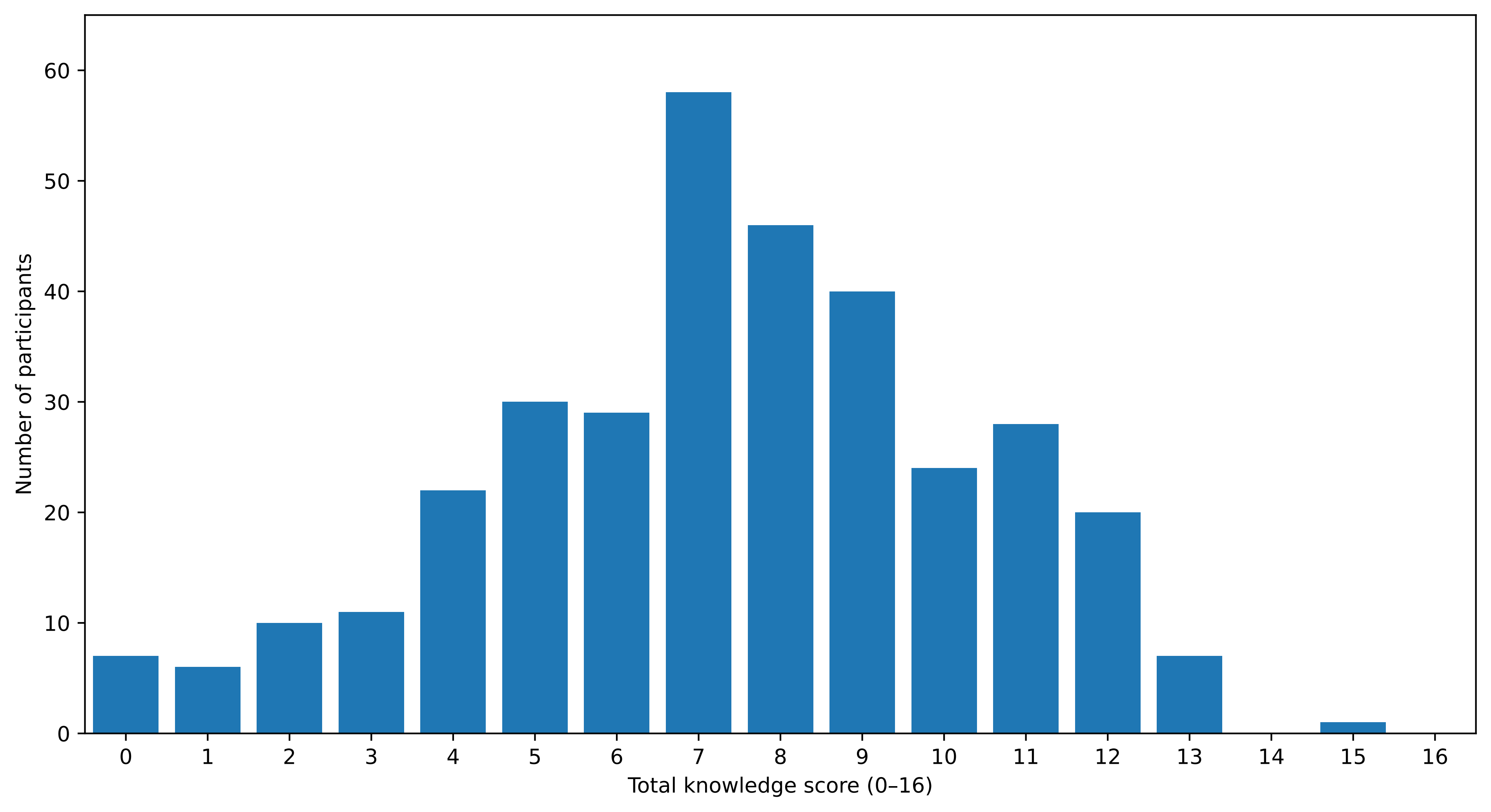

Number of participants
0
10
20
30
40
50
60
0
1
2
3
4
5
6
7
8
9
10
11
12
13
14
15
16
Total knowledge score (0–16)
