# Supplementary material for: Preparedness of undergraduate health students to provide women-specific epilepsy care: knowledge gaps and stigmatizing attitudes in Saudi Arabia
Source: Front Neurol. 2026 Oct 30;17:1983041. doi: 10.3389/fneur.2026.1983041 (PMC13759284; doi:10.3389/fneur.2026.1983041)
Supplement: Supplementary file 2 [file Data_Sheet_2.pdf]

Supplementary File S1. Complete Questionnaire

Examining Saudi Arabian Health College Students' Knowledge and Biases Regarding Women's Challenges with Epilepsy

Editorial note: *This supplementary file has been reformatted for publication. In the original Google Form, the knowledge items were phrased as questions with "Yes," "No," and "I do not know" response options. For publication, those items are presented as declarative statements with equivalent "True," "False," and "I do not know" labels to facilitate interpretation of the scoring key; the underlying item content and scoring were unchanged. The attitude section retains the wording shown to participants, including some non-person-first phrasing, while the main manuscript uses person-first language consistently. Google Forms interface elements have been removed.*

Participant information

Please remember that your participation is voluntary. You may withdraw from the study for any reason, at any time, without explanation, and without any sort of penalty. If you have any questions about the study, please contact Dr. Fahad Alzahrani at +966561130077 or fzahrani@taibahu.edu.sa. The College of Pharmacy, Taibah University Research Ethics Committee reviewed this study and granted ethics clearance on April 15, 2024. Should you have any questions regarding your rights as a participant in this study, you may email the Research Ethics Office at rec@taibahu.edu.sa. However, the final decision about participation is yours.

* Indicates a required question

Section 1: Demographic Information

1. What is your age? *

[ ] 18 [ ] 19 [ ] 20 [ ] 21 [ ] 22 [ ] 23 [ ] 24 [ ] 25 [ ] 26 [ ] 27 [ ] 28

2. What is your gender? *

[ ] Male [ ] Female

3. In which year of study are you currently? *

[ ] 1st year [ ] 2nd year [ ] 3rd year [ ] 4th year [ ] 5th year or above

4. What field are you studying? *

[ ] College of Pharmacy [ ] College of Medicine [ ] College of Dentistry [ ] College of Applied Medical Sciences [ ] College of Medical Rehabilitation Sciences [ ] College of Nursing [ ] Other

5. What is your current living arrangement? *

[ ] Living on-campus [ ] Living off-campus (e.g. with family) [ ] Other

6. Have you or any of your immediate family members been diagnosed with epilepsy? *

[ ] Yes [ ] No [ ] I'm not sure

7. Have you received any formal education or training on women's issues in epilepsy during your studies? *

[ ] Yes, I have covered this extensively [ ] Yes, but only briefly [ ] I'm not sure

8. When was the last time you, as a student, attended a training or educational session focused on women's issues in epilepsy? *

[ ] Within the past year [ ] 1–3 years ago [ ] More than 3 years ago [ ] I have never attended such a training [ ] I'm not sure

9. Would you be interested in participating in further education or training about epilepsy, particularly concerning its impact on women's health? *

[ ] Yes [ ] No

10. What resources do you typically refer to when needing information on women's issues in epilepsy? (Select all that apply) *

[ ] Textbooks/journal articles [ ] Clinical practice guidelines [ ] Online resources [ ] Consulting with more experienced colleagues [ ] I do not typically refer to any resources

Section 2: Knowledge regarding women's issues in epilepsy

Instructions: This section asks about knowledge regarding women's issues in epilepsy. Please select one response for each statement.

Item	Statement	True	False	I do not know
1	Epilepsy can be hereditary.	[ ]	[ ]	[ ]
2	Epilepsy is a form of mental illness.	[ ]	[ ]	[ ]
3	Convulsions can be a symptom of epilepsy.	[ ]	[ ]	[ ]
4	Loss of consciousness can be a symptom of epilepsy.	[ ]	[ ]	[ ]
5	Uncontrolled shouting is a typical symptom of epilepsy.	[ ]	[ ]	[ ]
6	Epilepsy can be controlled.	[ ]	[ ]	[ ]
7	Epilepsy is curable with modern medications.	[ ]	[ ]	[ ]
8	Surgery can be used to treat epilepsy.	[ ]	[ ]	[ ]
9	Epilepsy is more common in women than in men.	[ ]	[ ]	[ ]
10	Epilepsy can affect women's reproductive health, including menstrual cycles.	[ ]	[ ]	[ ]
11	Epilepsy can increase the risk of infertility in women.	[ ]	[ ]	[ ]
12	Some antiseizure medications taken during pregnancy may increase the risk of congenital malformations.	[ ]	[ ]	[ ]
13	Women with epilepsy require special considerations during pregnancy, including closer medication monitoring and management of seizure risk.	[ ]	[ ]	[ ]
14	Women with epilepsy may have an increased risk of postpartum depression.	[ ]	[ ]	[ ]
15	Hormonal and reproductive factors can influence the management and prognosis of epilepsy in women.	[ ]	[ ]	[ ]
16	Some antiseizure medications may reduce the effectiveness of hormonal contraceptives.	[ ]	[ ]	[ ]

Section 3: Attitudes towards women’s issues in epilepsy

Instructions: This section aims to understand health college students' attitudes towards women’s issues in epilepsy. Please indicate the extent to which you agree or disagree with each statement.

Item	Statement	Strongly agree	Agree	Neutral	Disagree	Strongly disagree
1	There are gender-specific challenges and considerations that need to be addressed when managing epilepsy in women	[ ]	[ ]	[ ]	[ ]	[ ]
2	Epileptic women can receive education.	[ ]	[ ]	[ ]	[ ]	[ ]
3	Epileptic women can perform daily activities.	[ ]	[ ]	[ ]	[ ]	[ ]
4	Epileptic women should participate in sports.	[ ]	[ ]	[ ]	[ ]	[ ]
5	A woman with epilepsy can have a job.	[ ]	[ ]	[ ]	[ ]	[ ]
6	A woman with epilepsy can marry.	[ ]	[ ]	[ ]	[ ]	[ ]
7	Women with epilepsy can have children.	[ ]	[ ]	[ ]	[ ]	[ ]
8	There is distress in the family when the mother suffers from epilepsy.	[ ]	[ ]	[ ]	[ ]	[ ]

Section 4: Stigma towards Women with Epilepsy

Instructions: Please indicate the extent to which you agree or disagree with each statement.

Item	Statement	Strongly agree	Agree	Neutral	Disagree	Strongly disagree
1	People with epilepsy are often viewed as less capable than others.	[ ]	[ ]	[ ]	[ ]	[ ]
2	I would feel uncomfortable socializing with someone who has epilepsy.	[ ]	[ ]	[ ]	[ ]	[ ]
3	Epilepsy is seen as a sign of weakness or lack of willpower.	[ ]	[ ]	[ ]	[ ]	[ ]
4	People with epilepsy are often discriminated against in the workplace.	[ ]	[ ]	[ ]	[ ]	[ ]
5	I would be hesitant to marry someone with epilepsy.	[ ]	[ ]	[ ]	[ ]	[ ]
6	Epilepsy is often misunderstood and misinterpreted by the general public.	[ ]	[ ]	[ ]	[ ]	[ ]
7	People with epilepsy are sometimes avoided or excluded from social activities.	[ ]	[ ]	[ ]	[ ]	[ ]
8	I would be concerned about the safety of my children around someone with epilepsy.	[ ]	[ ]	[ ]	[ ]	[ ]
9	Epilepsy is often associated with negative stereotypes and misconceptions.	[ ]	[ ]	[ ]	[ ]	[ ]
10	People with epilepsy sometimes face barriers in accessing education or employment opportunities.	[ ]	[ ]	[ ]	[ ]	[ ]

Prize draw registration

If you would like to register for the draw, please enter your university email.

4 people (100 riyals for each winner) by drawing participants in this survey.

University email: ____
