# Supplementary material for: Preparedness of undergraduate health students to provide women-specific epilepsy care: knowledge gaps and stigmatizing attitudes in Saudi Arabia
Source: Front Neurol. 2026 Oct 30;17:1983041. doi: 10.3389/fneur.2026.1983041 (PMC13759284; doi:10.3389/fneur.2026.1983041)
Supplement: Supplementary file 3 [file Data_Sheet_3.pdf]

Supplementary Table S1. Exploratory multivariable binary logistic regression of factors associated with meeting the ≥ 60% knowledge benchmark

Variable	B	SE	Wald	p-value	aOR	95% CI
**Gender**						
Male	−0.917	0.339	7.342	0.007*	0.400	0.206–0.776
Female (Ref.)						
**Academic year**			**11.347**	**0.023***		
First year	−0.852	0.469	3.308	0.069	0.426	0.170–1.068
Second year	−1.088	0.429	6.427	0.011*	0.337	0.145–0.781
Third year	−0.873	0.408	4.568	0.033*	0.418	0.188–0.930
Fourth year	−1.183	0.419	7.948	0.005*	0.307	0.135–0.697
Fifth year or more (Ref.)						
**Epilepsy diagnosis in self or immediate family**			**10.149**	**0.006***		
No	−0.995	0.534	3.477	0.062	0.370	0.130–1.052
Yes	0.019	0.594	0.001	0.974	1.019	0.318–3.266
I'm not sure (Ref.)						
**Formal education on women's epilepsy during studies**			**1.491**	**0.474**		
I'm not sure	−0.398	0.341	1.360	0.243	0.672	0.344–1.311
Yes, extensively	−0.260	0.439	0.350	0.554	0.771	0.326–1.823
Yes, briefly (Ref.)						
**Recent training on women's epilepsy issues**			**2.836**	**0.586**		
Within the past year	0.702	0.440	2.541	0.111	2.017	0.851–4.781
I'm not sure	0.248	0.377	0.433	0.511	1.282	0.612–2.684
1–3 years ago	0.454	0.478	0.905	0.341	1.575	0.618–4.018
More than 3 years ago	0.057	0.752	0.006	0.940	1.059	0.242–4.625
Never attended (Ref.)						

Model: $\chi^2(13) = 37.92$, $p < 0.001$; Cox & Snell $R^2 = 0.106$; Nagelkerke $R^2 = 0.159$; Hosmer–Lemeshow $\chi^2(8) = 8.02$, $p = 0.431$; AUC = 0.720; N = 339. aOR = adjusted odds ratio; CI = confidence interval; AUC = area under the receiver operating characteristic curve. $p < 0.05$. This analysis was exploratory because only 80 participants met the ≥60% benchmark relative to 13 estimated parameters (approximately 6.2 events per parameter); estimates should therefore be interpreted cautiously. Field of study was not included to limit model complexity given the small number of participants meeting the benchmark.
