# Supplementary material for: Preparedness of undergraduate health students to provide women-specific epilepsy care: knowledge gaps and stigmatizing attitudes in Saudi Arabia
Source: Front Neurol. 2026 Oct 30;17:1983041. doi: 10.3389/fneur.2026.1983041 (PMC13759284; doi:10.3389/fneur.2026.1983041)
Supplement: Supplementary file 4 [file Data_Sheet_4.pdf]

Supplementary Table S2. Pattern matrix from exploratory factor analysis of the stigma items

Stigma item	Factor 1: Personal stigma	Factor 2: Perceived societal stigma
I would feel uncomfortable socializing with someone who has epilepsy.	0.932	—
Epilepsy is seen as a sign of weakness or lack of willpower.	0.738	—
I would be concerned about the safety of my children around someone with epilepsy.	0.623	—
I would be hesitant to marry someone with epilepsy.	0.533	—
People with epilepsy are often discriminated against in the workplace.	0.467	0.402
People with epilepsy are often viewed as less capable than others.	0.463	0.331
People with epilepsy sometimes face barriers in accessing education or employment opportunities.	—	0.601
People with epilepsy are sometimes avoided or excluded from social activities.	—	0.538
Epilepsy is often associated with negative stereotypes and misconceptions.	—	0.506
Epilepsy is often misunderstood and misinterpreted by the general public.	—	0.476

Extraction method: principal axis factoring. Rotation method: Direct Oblimin with Kaiser normalization. Loadings <0.30 were suppressed. Kaiser–Meyer–Olkin (KMO) measure = 0.854; Bartlett's test of sphericity, $\chi^2(45) = 1056.29$, $p < 0.001$. The two-factor solution accounted for 43.9% of the extracted variance, and the correlation between Factors 1 and 2 was 0.346. Internal consistency among the four clearly loading items was $\alpha = 0.794$ for Factor 1 and $\alpha = 0.649$ for Factor 2. The perceived-capability and workplace-discrimination items cross-loaded on both factors and were therefore interpreted cautiously.
